# Cellular Performance Comparison of Biomimetic Calcium Phosphate Coating and Alkaline-Treated Titanium Surface

**DOI:** 10.1155/2013/832790

**Published:** 2013-12-24

**Authors:** Xiaohua Yu, Mei Wei

**Affiliations:** Department of Materials Science and Engineering, University of Connecticut, 97 North Eagleville Road, Unit 3136, Storrs, CT 06269, USA

## Abstract

The influence of biomimetic calcium phosphate coating on osteoblasts behavior *in vitro* is not well established yet. In this study, we investigated the behavior of osteoblastic rat osteosarcoma 17/2.8 cells (ROS17/2.8) on two groups of biomaterial surfaces: alkaline-treated titanium surface (ATT) and biomimetic calcium phosphate coated ATT (CaP). The cell attachment, proliferation, differentiation, and morphology on these surfaces were extensively evaluated to reveal the impact of substrate surface on osteoblastic cell responses. It was found that the ROS17/2.8 cells cultured on the ATT surface had higher attachment and proliferation rates compared to those on the CaP surface. Our results also showed that the calcium phosphate coatings generated in this work have an inhibiting effect on osteoblast adhesion and further influenced the proliferation and differentiation of osteoblast compared to the ATT surface *in vitro*. Cells on the ATT surface also exhibited a higher alkaline phosphatase activity than on the CaP surface after two weeks of culture. Immunofluorescence staining and scanning electron microscopy results showed that the cells adhered and spread faster on the ATT surface than on the CaP surface. These results collectively suggested that substrate surface properties directly influence cell adhesion on different biomaterials, which would result in further influence on the cell proliferation and differentiation.

## 1. Introduction

Titanium and its alloys have been used for orthopedic applications for decades because of their excellent mechanical properties, superior biocompatibility, and good corrosion resistance [1]. However, these titanium-based materials also suffer from drawbacks, such as insufficient bioactivity which leads to poor osseointegration of the implant with host bone [2]. Tremendous efforts have been made to optimize the surface property of titanium such as surface chemistry, composition, and topography in order to improve the bioactivity of Ti implants and accelerate bone healing [3–5]. For instance, calcium phosphate (CaP) has been coated on Ti implant surfaces to provide the implants with superior osteoconductivity due to the physiochemical property of CaP ceramics [6–8]. A variety of CaP coating technologies have been developed, such as plasma splaying, sputtering deposition, sol-gel coating, and ion implantation. They have been used to obtain CaP coatings on Ti implant surfaces [9–11]. Recently, an approach in creating biomimetic coating has attracted substantial interests of researchers due to its simplicity, flexibility, and low cost [12–15]. A bone-mineral-like CaP coating can be achieved on titanium surface by simply incubating the implants in modified simulated body fluid (m-SBF) at ambient conditions [16]. Many studies have demonstrated that the biomimetic CaP coating could actively promote bone ingrowth and improve implant-bone integration [17–21].

Biomimetic CaP coatings have shown their impact on regulating diverse cell behaviors. However, researchers from different groups obtained conflicting results regarding CaP coating-cell interactions. Most of the earlier studies supported that CaP coating improved osteoblast adhesion, proliferation, and differentiation *in vitro*, as well as accelerated bone growth, bone matrix apposition, and biominerilization process *in vivo *[22–25]. However, more recent reports showed some conflicting results that CaP coating suppressed the activity of osteoblasts such as lowering cell proliferation and reducing cell differentiation [26, 27]. Lee and his colleagues demonstrated that osteoblasts had a lower proliferation rate on apatite surface compared to tissue culture dish [28]. Murphy et al. suggested that bone-like mineral accelerated cell proliferation and growth but inhibited cell differentiation with a lower osteogenic marker expression [29]. The diverse influence of CaP coating on osteoblasts behavior may be attributed to its materials characteristics, such as surface topography, composition, crystallinity, crystal size, and dissolution rate [30, 31]. When biomimetic CaP coating is applied on biomaterial surfaces, it does not only change the topography of the original surface, but also changes the chemical composition which interacts directly with cells [32]. Although the effect of surface topography on cell responses has been extensively investigated, the influence of the combination of chemical surface modification and designed topography on cell responses still remains unexplored.

In this study, two groups of biomaterial surfaces: alkaline-treated titanium surface (ATT) and biomimetic calcium phosphate coated titanium surface (CaP) were employed to represent two types of materials surfaces with distinct surface topography and chemical composition. Osteoblastic cell line ROS17/2.8 was used to systemically investigate the impact of substrate signals on cellular responses. In particular, the cell adhesion behavior on the tested material surfaces was correlated with the long-term cell growth and differentiation to reveal the relationship between biomaterial surface and implant performance.

## 2. Materials and Methods

### 2.1. Preparation and Characterization of Biomimetic CaP Coating

Biomimetic CaP coatings were prepared on titanium substrates using a method described in earlier studies [24, 33]. Commercially available pure titanium strips (10 mm × 10 mm × 0.2 mm) were used in the current study. They were roughened using 800 sandpapers, followed by alkaline treatment in 5 M NaOH at 60°C for 24 h. All the samples were then thoroughly washed with deionized water and dried in air at room temperature. Half of the samples were reserved at this point for cell culture study. The treated titanium foils were soaked in modified simulated body fluid (m-SBF) (6.0 mM NaCl, 3.0 mM K_2_HPO_4_·3H_2_O, 3.0 mM MgCl_2_·6H_2_O, 50 mM HEPES, 8.0 mM CaCl_2_, 18 mM NaHCO_3_) to achieve a layer of bone-like apatite coating. The plates were immersed in the m-SBF at 42°C for 24 h. After the coating process, all coated titanium plates were rinsed with deionized water and dried at room temperature. Two groups of materials were used for cell culture in the subsequent study: alkaline-treated pure titanium plates (ATT) and alkaline-treated and biomimetic CaP coated titanium plates (CaP). All the plates were sterilized at 121°C for 55 min before cell culture.

### 2.2. Surface Characterization

**Figure 1:**
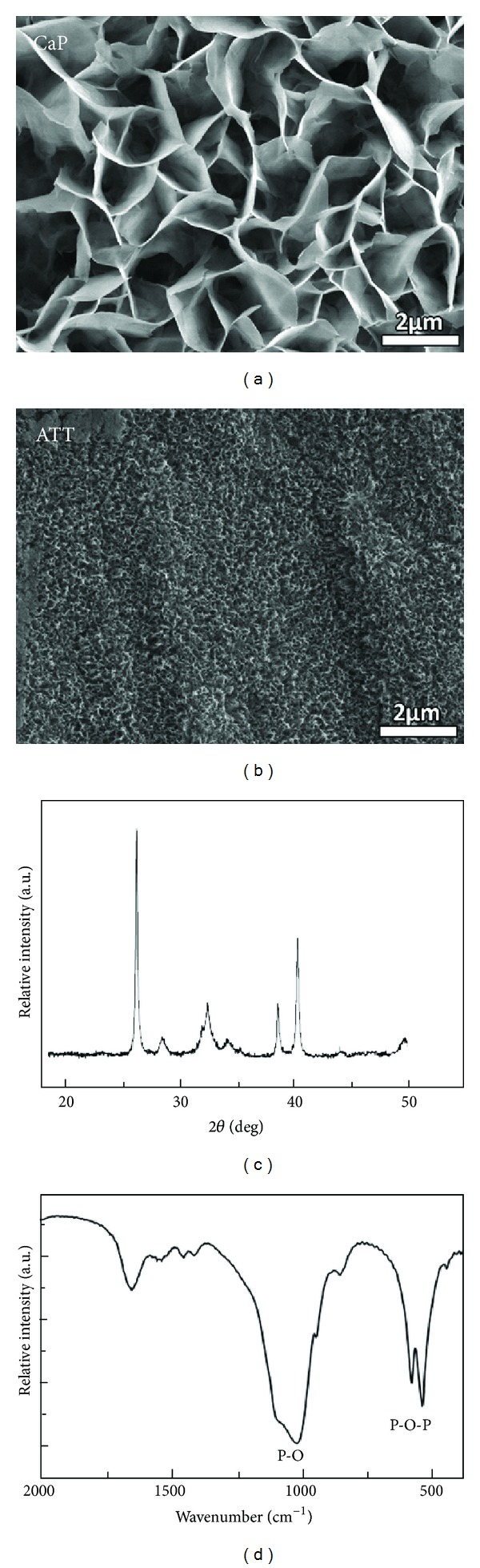
Characterization of different substrates. Upper panel: CaP: FESEM micrograph of biomimetic calcium phosphate surface. ATT: FESEM micrograph of alkaline-treated titanium surface. Lower level: X-ray diffraction pattern of CaP. FTIR spectrum of the CaP.

The surface morphology of the two tested surfaces was observed using field emission scanning electron microscopy (FESEM, JEOL6335F) at 5 kV (see Figure 1). CaP coating was also examined using X-ray diffractometer (Bruker AXS D5005) with a copper target. The voltage and current setups were 40 kV and 40 mA, respectively. Plus, the CaP coating was also evaluated using Fourier transform infrared spectroscopy (FTIR, Nicolet Avatar 360). The FTIR spectrum was recorded in the range 400–2000 cm^−1^.

### 2.3. Cell Culture

Rat osteosarcoma ROS17/2.8 cells were cultured in F12 medium supplemented with 10% FBS (Cellgro, USA) and 1% pen-strep (Cellgro, USA). Cells were grown in a humidified atmosphere of 5% CO_2_ at 37°C. Culture media were changed every other day. An osteogenic medium, which consists of F12 plus 10 mM *β*-glycerol phosphate, 10 nM dexamethasone, and 50 *μ*g/mL L-ascorbic acid (Sigma, St. Louis, MO), was used after the cells were grown on the materials for one week.

### 2.4. Cell Attachment

ROS17/2.8 cells were seeded on ATT and CaP plates in a 24-well plate at a density of 2 × 10^4^ cells/cm^2^ in 1.0 mL medium (*n* = 5). The cells were allowed to attach on the test surfaces for 4 h before all culture medium was aspirated from each well. The samples were then washed by PBS three times to remove the unattached and loosely bound cells from material surfaces. An Alamar Blue assay was chosen to measure the density of cells left on the samples. 0.5 mL fresh medium containing 10% Almar Blue dye (Biosource International, USA) was added to each well and incubated for 2 h. The incubated medium was then transferred into a 96-well plate and read by a microplate reader (Biotek MQX, USA) at absorption wavelengths of 570 and 600 nm. The cell numbers on different substrates were calculated based on a calibration curve with known amount of cells in each well.

### 2.5. Cell Proliferation

ROS17/2.8 cells were seeded onto ATT and CaP plates in a 24-well plate at a density of 2 × 10^4^ cells/cm^2^ in 1.0 mL medium (*n* = 5). The medium was refreshed every two days. After 3, 7, and 14 days of incubation, the cell numbers on different substrates were measured using the Alamar Blue assay as described in Section 2.3. At each time point, 200 uL 10% Alamar Blue in culture medium was added into each well after aspirating the existing medium. After incubating at 37°C for 2 h, 100 uL of the solution was transferred from each well into a 96-well plate and ready at *λ*_ex_/*λ*_em_ = 570/600 nm The results were expressed as relative cell number compared with the control (cell number on ATT) at day 3.

### 2.6. Cell Differentiation

The activity of alkaline phosphatase (ALP) was measured as described previously [33]. ROS17/2.8 cells were seeded and cultured in the same way as in the proliferation study. Cell differentiation ability was evaluated at days 3, 7, and 14 (*n* = 5). To measure ALP activity, the cells were washed with PBS and lysed with 0.2 mL 0.5% Triton X-100 in PBS. The lysis was sonicated for 60 s and centrifuged at 5 × 10^3^ RPM 4°C for 10 minutes. Aliquots of supernatants were subjected to a total protein assay using a BCA assay kit (Pierce, USA). The ALP activity was measured by colorimetry in ALP assay reagent mixture composed of 5 mM *p*-nitrophenol phosphate disodium (*p-*NPP), 1 mM MgCl_2_, and 0.15 M 2-amino-2-methyl-1-propanol (AMP) (Sigma, USA) with an equal volume amount of nitrophenyl phosphate (10 mM). The absorbance was measured at 405 nm using a *μ*Quant microplate reader (*μ*Quant, Bio-Tek, USA). The ALP activity was expressed as per microgram total protein for each sample.

### 2.7. Immunofluorescence Staining

ROS 17/2.8 cells were seed on ATT and CaP plates in a 24-well plate at a final density of 1.0 × 10^4^ cells/cm^2^ for 12 and 24 h. At each time point, the specimens were rinsed with PBS. They were then fixed with 4% formaldehyde in PBS for 20 min at room temperature, permeabilized in 0.5% Triton X-100 in PBS for 15 min, and finally incubated with 1% BSA in PBS for 1 h at room temperature. Antivinculin antibody (Sigma, USA) was diluted at a ratio of 1 : 128 and incubated with the cells for 1 h at 37°C. After thorough rinses using PBS, the specimens were incubated with a goat-anti-mouse-IgG-FITC-conjugated secondary antibody (1 : 150, Sigma, USA). To detect actin and nucleus simultaneously, tetramethylrhodamine isothiocyanate- (TRITC-) conjugated phalloidin (1 : 400, Invitrogen, USA) and 0.5 *μ*g/mL 4′,6-diamidino-2-phenylindole dihydrochloride (DAPI) were added in the secondary antibody solution. Triple-stained cells were observed using a fluorescent microscope (Zeiss Axiovert 200 M) with filters appropriate for FITC, TRITC, and DAPI.

### 2.8. Cell Morphology

Cells were seeded on ATT and CaP plates in a 24-well plate at a density of 1.0 × 10^4^ cells/cm^2^ for 6, 12, 24, and 48 h, respectively. After culture, the cells were fixed in 2.5% glutaraldehyde buffer for 1 h and incubated in 0.1 M sodium cacodylate buffer for another hour. The fixed cells were then dehydrated in graded ethanol series and followed by a critical point drying. All the samples were sputter-coated with gold palladium. Finally, the cell morphology on different substrates was examined using field emission scanning electron microscopy (FESEM, LEO/Zeiss DSM 982). To assess the cell distribution and extracellular matrix deposition, cells were seeded and cultured on ATT and CaP plates for 2 weeks. After that, all the specimens were prepared as described earlier in this section and then subjected to FESEM observations.

### 2.9. Statistical Analysis

All the data were illustrated as the mean ± standard deviations. The statistical difference was analyzed using analysis of variance (ANOVA), and *P* < 0.05 was considered significant.

## 3. Results

### 3.1. Surface Characterization

The morphology of the two tested surfaces showed distinct differences. The CaP coating surface demonstrated a plate-like structure uniformly covering the titanium surface. The size of these plate-like structure is around 3-4 *μ*m. In comparison, the ATT surface exhibited a porous network associated with a nanometer sized structure in a scale of approximately 100–200 nm. The XRD spectrum of CaP showed a cluster of peaks around 31–33° which are assigned to (211), (112), and (300) planes of hydroxyapatite. The FTIR spectrum showed characteristic bands at 1040, 602, and 563 cm^−1^ which could be assigned to P-O stretching and O-P-O bending mode. These data collectively suggest that the CaP coating is poorly crystalline apatite.

### 3.2. Cell Attachment and Proliferation

**Figure 2:**
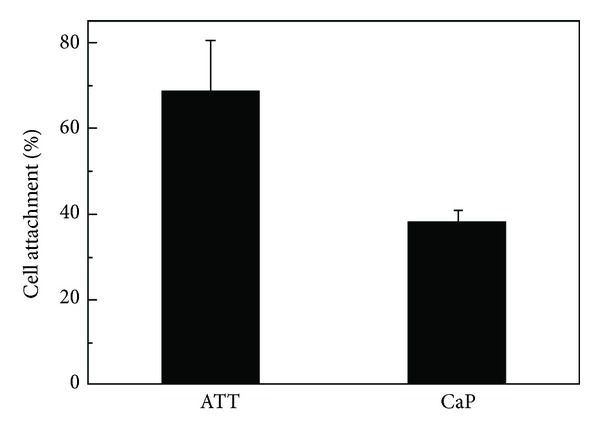
Cell attachment on different substrates measured by the Alamar Blue assay. Incubated for 4 h, significantly more cells (*P* < 0.05) were attached to the surface of ATT than CaP.

Cell attachment on tested material surfaces was assessed by performing a short time cell adhesion assay.  Figure 2 shows ROS17/2.8 cell attachment expressed as a percentage of total cells seeded on ATT and CaP surfaces. The seeded cells successfully attached to both ATT and CaP surfaces after 4 h incubation, but the number of cells on each type of material appeared to be substantially different. ROS17/2.8 cells seed onto the ATT surface (69%, *P* < 0.05) exhibited significantly higher cell attachment than that of CaP (38%, *P* < 0.05), which indicates that the cells might have attached to the ATT surface faster than that of the CaP.

**Figure 3:**
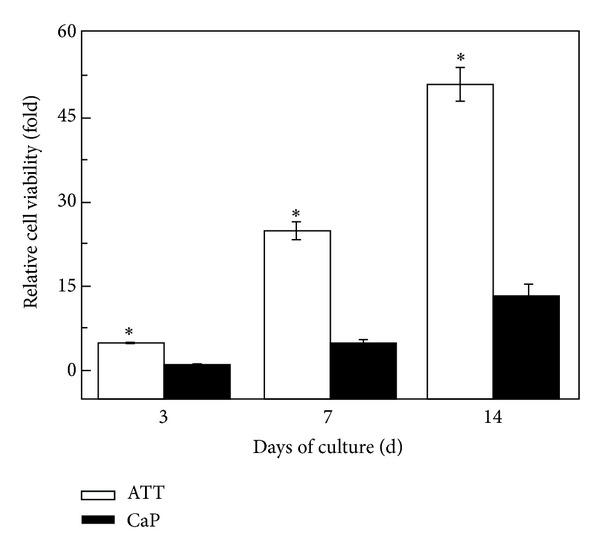
Proliferation of osteoblastic cells cultured on ATT and CaP surfaces for 3, 7, and 14 days. The cell proliferation was significantly higher on ATT than on CaP (*P* < 0.05) at all time points.

The cell proliferation was expressed as the number of living cells present on both groups of surfaces at day 3, 7, and 14 of culture (Figure 3). The cell number increased steadily on both materials as the culture time extended. At day 3, the cell number on ATT was significantly higher than that on CaP (*P* < 0.05). At day 7 and 14, the difference of cell numbers between these two groups became more significant. The cell number on ATT at day 14 was almost 4-fold of that on CaP at this time point. Besides, the cell generation time of ROS17/2.8 cells was also calculated based on the following equation:

(1) $$\begin{array}{c} N_{t}=N_{0}2^{tf}, \end{array}$$

where *N*_0_ is the initial cell number, *N*_*t*_ is the cell number after *t* days of culture, *t* is the culture period, and 1/*f* is the generation time (h/generation).

It was found that although the cell number on CaP was much lower than on ATT, there is no significant difference between cell doubling times from day 3 and day 7 (Table 1) for these two surfaces. Surprisingly, the cell doubling time on CaP is 133 h between days 7 and 14, which is significantly shorter than that of ATT (162 h).

### 3.3. Cell Differentiation

**Figure 4:**
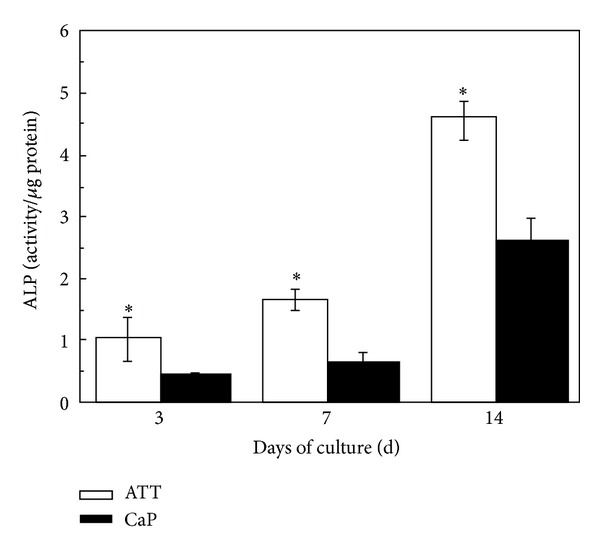
ALP activity normalized to total protein content of osteoblastic cells cultured on ATT and CaP surfaces for 3, 7, and 14 days. ALP activities were significantly higher on ATT than on CaP (*P* < 0.05) at all time points.

Alkaline phosphatase (ALP) activity is one of the most widely used marker for early differentiation of osteoblasts [34]. Although the ALP level on both surfaces started low in the first week, it dramatically increased in the second week of culture. Importantly, it was noticed that the ALP activity of the cells on ATT group was higher than on CaP at all time points. However, the ALP increase rate of the two groups was very similar. While ALP activity of the ATT group at days 3 and 7 was more than two times higher than the CaP group, it dropped to only onefold higher than that of CaP at day 14 (see Figure 4).

### 3.4. Immunofluorescence Staining

**Figure 5:**
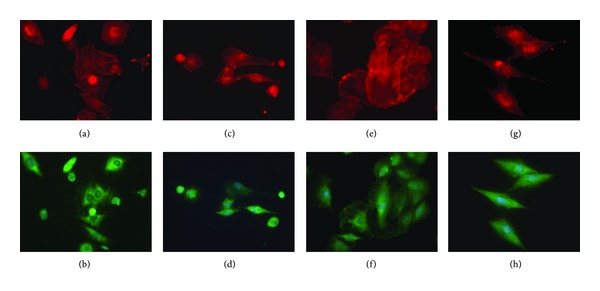
Immunostaining of vinculin, actin, and nuclei showing ROS17/2.8 osteoblastic cells cultured on ATT and CaP surfaces for 24 and 48 h, respectively. (a) and (b) ATT at 24 h, (c) and (d) CaP at 24 h, (e) and (f) ATT at 48 h, and (g) and (h) CaP at 48 h. Green: vinculin; Red: actin; Blue: nuclei.

Immunocytochemistry was conducted to evaluate cell adhesion on both groups of materials (Figure 5). Cells plated on ATT surface spread out well and organized actin into stress fibers after 12 h, while the cells on CaP surface showed a more round shape and failed to form stress fibers (Figures 5(a) and 5(c)). After 24 h of incubation, the cells on ATT reached full spreading and developed a distinct focal adhesion (Figure 5(f)). Focal contact clusters were readily found near the periphery of cells. In contrast, the cells on CaP illustrated a more slim and elongated shape which indicates insufficient spreading of the cells (Figures 5(b) and 5(d)). There was no focal contact clusters formed in the cells on CaP (Figure 5(h)).

### 3.5. Cell Morphology

**Figure 6:**
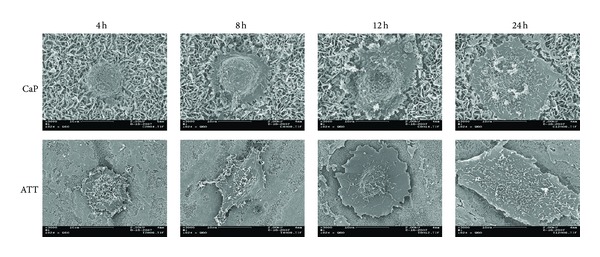
SEM micrographs showing ROS17/2.8 osteoblastic cell morphologies on ATT and CaP surfaces for different time periods. Scale bar = 10 *μ*m.

Figure 6 shows the morphology of ROS17/2.8 cells cultured on ATT and CaP surfaces at different time points. SEM micrographs were taken at 4, 8, 12, and 24 h to record the complete cell adhesion process on different substrates. After cells were seeded on the substrates for 4 h, the cells on CaP demonstrated a round morphology while those on ATT were spread out. At 8 h, the cells on CaP also began to spread with some short filopodia formed around the cellular body. In contrast, the cells on ATT stretched to a great extent and long filopodia were found anchoring to the material surface. The cells on CaP kept expanding and became much flatter in the next four hours while the cells on ATT almost reached a full degree of spreading. After culturing for 24 h, the cells on CaP finally displayed a complete spreading and developed a good adhesion to the underlying surface, while the cells on ATT also attached closely to the surface but with a larger contacting area.

**Figure 7:**
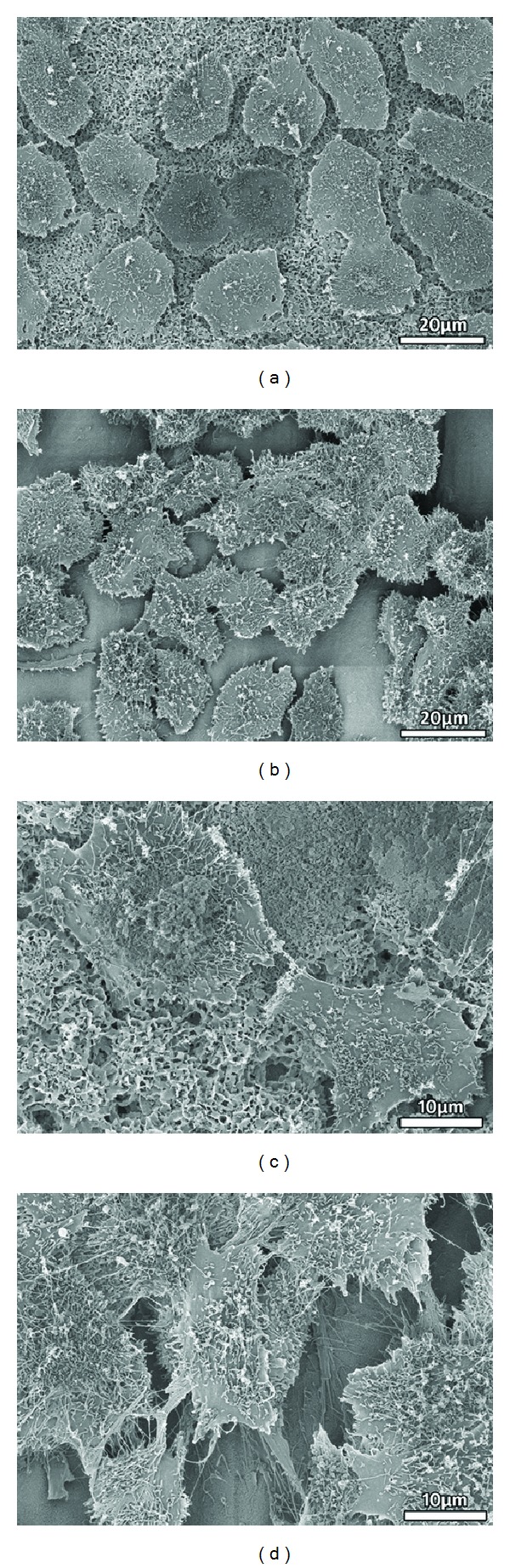
SEM micrographs showing ROS17/2.8 osteoblastic cell morphologies cultured on the ATT and the CaP coating surfaces for 14 days. (a) CaP (at low mag), (b) ATT (at low mag), (c) CaP (at high mag), and (d) ATT (at high mag).

Figure 7 shows the ROS17/2.8 cells growing on the two types of surfaces after 14 days of culture. Both surfaces were covered uniformly with a layer of cells. In particular, cells on ATT grew tightly to each other and tended to form cell colonies (Figure 7(b)). Cells on CaP grew more sparsely instead of forming tight contact with each other (Figure 7(a)). At a high magnification, numerous filopodia were observed on cells grown on ATT, but less filopodia were observed in cells on CaP (Figures 7(c) and 7(d)).

## 4. Discussion

Biomaterial surfaces play a vital role in tissue engineering and regenerative medicine because most biological reactions during implantation occur between the implant surface and the biological environment [35]. Calcium phosphate coatings on implant surfaces have been employed to improve the performance of implants through enhanced osteoblastic cell activities, such as cell proliferation, differentiation, and mineral deposition on the implants [36, 37]. However, recent studies also reported conflicting results of the impact of CaP coating on osteoblastic cells [28, 29]. In this work, we aimed at correlating the cell adhesion behavior with long-term cellular performance on two types of biomaterial surfaces in order to illustrate the critical role of CaP coating to cellular responses. We found that osteoblastic attachment and adhesion were weakened on the so-prepared CaP coating surface compared to the alkaline-treated ATT surface. As a result, osteoblasts proliferate and differentiate on the CaP surface were significantly delayed and impaired. Our results indicate that osteoblast-biomaterial interactions are significantly affected by substrates surface properties [38].

The adhesion of osteoblast on biomaterials mainly depends on the surface properties of materials such as topography, chemistry, and composition [39–41]. Osteoblasts seeded on ATT demonstrated better organized actin and more focal adhesion compared to those on CaP. SEM observations also exhibited that cell attachment on ATT is faster than on CaP (Figures 5 and 6). It has been reported that nanometric topography of a biomaterial had a significant impact on cell adhesion [42]. Surface characterization of the two tested groups showed distinct topographical features on ATT and CaP: formation of the CaP on titanium substrate resulted in micrometric topography, while alkaline-treated titanium substrate demonstrated nanometric topography. This distinction of surface topography substantially affected the follow-up *in vitro* tests such as protein adhesion, focal plaques formation, and cell spreading [43]. Protein adsorption on a nanometric topographical titanium substrate was found to be much higher and more oriented compared to a micrometric titanium surface [44]. Thus, the ATT might enable more adhesive protein adsorption such as fibronectin and laminin from the serum. Besides, focal plaque mediated cell adhesion was also enhanced by the presence of nanotopography on biomaterials surface [45]. For instance, Okada et al. found that focal adhesion could only be formed on hydroxyapatite surface with nanoscale feature but not on a smooth dense surface [46]. More cell adhesion receptors could have been activated when there were adequate interactions between cells and ATT, a substrate with high surface energy, resulting in earlier cell adhesion [46]. In contrast, the slender cell shape and delayed cell adhesion on the CaP substrate indicated that the cells did not interact well with the substrate (Figures 5 and 6). Therefore, both the literature and our observations suggest the unique nanotopography of the ATT surface might have played a crucial role in cell adhesion in this context.

Cell adhesion influences many aspects of cell behavior, including proliferation, differentiation, morphology, and migration [47]. There are studies showing that cell membrane in contact with the nanostructured topography was subject to tensile and relaxation mechanical forces that trigger cell behavior in certain ways [48]. Recent research has shown that signaling pathways triggered by growth factors require strong cell adhesion for cell cycle progression and proliferation [49]. The high cell proliferation rate of ROS17/2.8 cells on ATT may be due to their better adhesion on the surface (Figures 5 and 6). As a result, cell cycle phase progression and proliferation on ATT are triggered earlier than CaP. Lee and colleagues also reported similar results that CaP coating had negative influence on cell proliferation due to its provision of insufficient adhesion signals [26]. It was unexpected that the cell doubling time of cells on CaP is shorter than on ATT during the second week of culture. A possible explanation is that the negative influence of weak adhesion is only present in the early stage of cell growth. Once the cell adhesion is completed, cell proliferation may not be affected by the adhesion as much as the initial stage. This might also explain the ALP result. Although the ALP activity on CaP is much lower than that of ATT at the initial stage, the ALP activity increased 4-folds on CaP in the second week, while it only increased 2.78-folds on ATT. It is likely that the negative effect of poor adhesion on CaP gradually faded out in the second week and the other positive aspects of CaP coating such as Ca^2+^ release pushed the CaP coating to catch up on cell differentiation.

Although *in vitro* cell culture results have provided useful information for initial-stage biological screening of biomaterials, the data from cell culture cannot yet be fully correlated with *in vivo* implant performances. Some recent published studies have shown inhibitory effect of calcium phosphate coatings on osteoblasts* in vitro *[50, 51], but their performance *in vivo* cannot be completely predicted by these data. It is noteworthy that most of these studies showing negative influence of calcium phosphate coating were based on cell culture study. Better cell adhesion on titanium *in vitro* does not necessarily suggest ATT demonstrates better biological properties than CaP *in vivo*. Distinct differences have been found between titanium and calcium phosphate when they were implanted into animal bodies. Compared to titanium which is basically inert in the body, calcium phosphate is bioactive during the bone healing. Calcium phosphate provides direct bone contact at the implant-bone interface and guide bone formation along their surfaces by formation of a biological apatite layer [52]. In the case of titanium, macrophages often show up adjacent to the titanium implants which did not have direct bone bonding [53, 54]. Although surface treated titanium showed better cellular interactions than biomimetic CaP coating *in vitro* in this work, when they are applied to the physiological environment, the tissue reaction may vary substantially. Besides, the long-term performance of titanium has raised certain concerns due to corrosion and formation of wear debris [3]. Thus, it is critical to notice that the cell culture model might provide useful information for biomaterials screening; the merit of the biomaterials can only be confirmed in animal models and other clinical trials.

## 5. Conclusion

In this study, the cellular responses to biomimetic calcium phosphate coating were systematically investigated in comparison with an alkaline-treated titanium surface. It was found that the calcium phosphate coating used in this work had an inhibiting effect osteoblast proliferation and differentiation. The inhibitory impact of the calcium phosphate coating might be caused by the poor adhesion of cells at the initial stage of cell-surface interactions. Thus, the results of this study collectively highlights that cellular performance of biomaterials might be varied by multiple material surface properties such as composition, topography, surface energy and other related factors.

## Figures and Tables

**Table 1:** ROS17/2.8 generation time (*1/f*) on ATT and CaP at different time periods.

Generation time (1/*f*)	Day 7	Day 14
ATT	41.61 ± 1.89	162.23 ± 2.86
CaP	41.79 ± 3.61	133.23 ± 5.93
